# Two Cases of *Borrelia miyamotoi* Meningitis, Sweden, 2018

**DOI:** 10.3201/eid2510.190416

**Published:** 2019-10

**Authors:** Anna J. Henningsson, Hilmir Asgeirsson, Berit Hammas, Elias Karlsson, Åsa Parke, Dieuwertje Hoornstra, Peter Wilhelmsson, Joppe W. Hovius

**Affiliations:** Linköping University, Linköping, Sweden (A.J. Henningsson, P. Wilhelmsson);; County Hospital Ryhov, Jönköping, Sweden (A.J. Henningsson, P. Wilhelmsson);; Karolinska Institutet, Stockholm, Sweden (H. Asgeirsson);; Karolinska University Hospital, Stockholm (H. Asgeirsson, B. Hammas, Å. Parke);; Danderyd University Hospital, Stockholm (E. Karlsson);; Academic Medical Center, Amsterdam, the Netherlands (D. Hoornstra, J.W. Hovius)

**Keywords:** *Borrelia miyamotoi*, meningitis, PCR, serology, Sweden, bacteria

## Abstract

We report 2 human cases of *Borrelia miyamotoi* disease diagnosed in Sweden, including 1 case of meningitis in an apparently immunocompetent patient. The diagnoses were confirmed by 3 different independent PCR assays and DNA sequencing from cerebrospinal fluid, supplemented by serologic analyses.

*Borrelia miyamotoi* is the cause of an emerging disease in the Northern Hemisphere, transmitted by hard (*Ixodes*) ticks. The bacterial species, described in Japan in 1995 (*1*), is genetically related to the relapsing fever borreliae and may be divided into Siberian, European, and American genotypes (*2*). *B. miyamotoi* disease (BMD), described in case series from Russia (*3*) and the United States (*4*,*5*), is a systemic illness causing relapsing fever, headache, myalgia, arthralgia, elevated liver enzymes, neutropenia, and thrombocytopenia. In addition, 3 cases of meningoencephalitis caused by *B. miyamotoi* have been reported worldwide, 2 from Europe and 1 from the United States, all in highly immunocompromised patients (*6*–*8*). We report 2 human cases of BMD diagnosed in Sweden, including 1 case of meningitis in an apparently immunocompetent patient.

## The Patients

**Figure 1:**
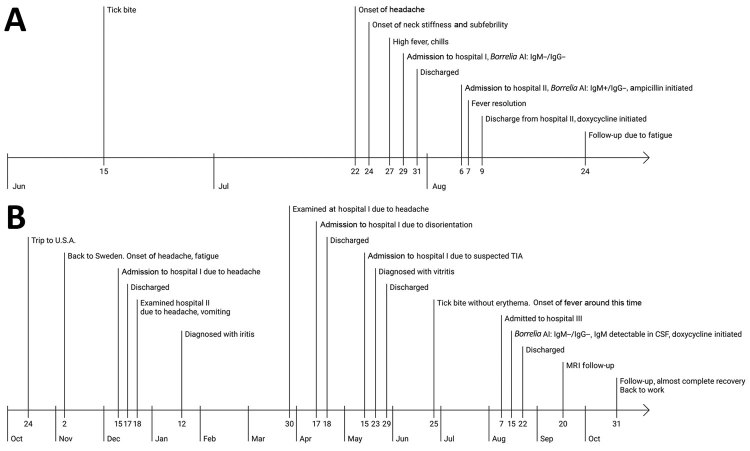
Time course of *Borrelia miyamotoi* meningitis in 2 patients, Sweden, 2018. A) Patient A, a 53-year-old immunocompetent woman; B) patient B, a 66-year-old immunocompromised woman. AI, antibody index; CSF, cerebrospinal fluid; MRI, magnetic resonance imaging; TIA, transient ischemic attack.

On July 29, 2018, a 53-year-old woman (patient A) sought care at a hospital for headache, neck stiffness, and high-grade fever that had progressively worsened during the preceding week (Figure 1, panel A). Her medical history included previous cholecystectomy and gastric bypass surgery. Her sole medication was oxycodone (an opioid drug used to manage pain), which she was taking because of a recent elbow fracture. She had not been abroad during the preceding months but she had removed an attached tick while staying in Stockholm County 6 weeks earlier. No subsequent erythema appeared around the bite site. At admission, we found no neurologic deficits or signs of impaired consciousness. Cerebrospinal fluid (CSF) analysis showed total leukocyte count 321 cells/μL (reference <5 cells/μL), mononuclear cells 276 cells/μL (reference <5 cells/μL), and albumin 1,270 mg/L (reference <420 mg/L). Bacterial culture; anti-*Borrelia* antibody testing; and PCR for herpes simplex virus, varicella zoster virus, and enterovirus were negative in CSF. Serologic test results for tickborne encephalitis were negative. Viral meningitis was suspected. 

The next day, clinical improvement occurred, and the patient was discharged. However, the patient’s condition then worsened, with more pronounced headache and neck pain, and she was readmitted on August 6. Blood cell and platelet counts and C-reactive protein levels were normal. CSF analysis showed total leukocyte count 517 cells/μL (reference <5 cells/μL), mononuclear cells 354 cells/μL (reference <5 cells/μL), and CXCL13 327 pg/mL (reference <190 pg/mL). We initiated intravenous treatment with ampicillin to cover *Listeria* meningitis; the fever resolved within 1 day. The CSF *Borrelia* antibody index came back weakly positive for IgM (Table) and, under the diagnosis of (atypical) Lyme neuroborreliosis (LNB), oral doxycycline was initiated (200 mg 2×/d for 14 d). Panbacterial *16S* rRNA gene sequencing (*9*) of the CSF sample suggested *B. miyamotoi*, a finding that later was confirmed by specific PCR, sequencing, and serologic testing (Table).

**Table:** Confirmatory analyses performed on cerebrospinal fluid and serum/plasma samples from 2 patients with diagnoses of meningitis caused by *Borrelia miyamotoi*, Sweden, 2018*

Analysis	Patient A, dates samples collected								Patient B, dates samples collected				
	Jul 30			Aug 6			Aug 24		Aug 9				Oct 31
Sample	Serum	CSF		Serum	CSF		Serum		Serum	Plasma	CSF		Serum
BM-specific PCR	–	+		+	+		NA		NA	+	+		NA
BM-specific serologic testing	IgM+ (GlpQ, Vlp5), IgG–	NA		IgM+ (GlpQ, Vlp5), IgG+ (GlpQ)	NA		IgM+ (GlpQ, Vsp1), IgG+ (GlpQ, Vlp15/16)		IgM+ (GlpQ), IgG–	NA	NA		IgM+ (GlpQ), IgG–
LB serology†	–	–		–	IgM AI+		–‡		–	NA	IgM detectable, but AI–		–‡
Culture attempts	–	–		–	–		NA		NA	–	NA		NA

*Patient A: a 53-year-old immunocompetent woman; patient B: a 66-year-old immunocompromised woman. AI, antibody index; BM, *Borrelia miyamotoi*; CSF, cerebrospinal fluid; GlpQ, glycerophosphodiester-phosphodiesterase; LB, Lyme borreliosis; NA, not analyzed; Vlp, variable large protein; Vsp, variable small protein; +, positive; –, negative. †LIAISON *Borrelia burgdorferi* (DiaSorin, https://www.diasorin.com). ‡In addition to LIAISON, the sample was analyzed by Enzygnost Lyme link VlsE IgG and Enzygnost Borreliosis IgM (DADE Behring, https://www.siemens.com), recomBead Borrelia IgM and IgG (Mikrogen GmbH, https://www.mikrogen.de), C6 Lyme ELISA Kit (Immunetics, https://immunetics.com), and Anti-Borrelia EUROLINE-RN-AT IgG and IgM (EUROIMMUN, http://www.euroimmun.com). All test results were negative.

At follow-up on August 24, the patient showed continued improvement without any fever relapses. Complementary tests for immunodeficiency showed normal serum levels of immunoglobulins. We analyzed convalescent serum using several commercially available tests for laboratory diagnosis of Lyme borreliosis (Table); all results were negative.

A 66-year-old woman (patient B) living in Stockholm County was referred in August 2018 for 6 weeks of intermittent high-grade fever and 9 months of various other symptoms (Figure 1, panel B). She had rheumatoid arthritis, treated with methotrexate together with rituximab twice a year since 2011, but had been physically very active. Her symptoms began with headache and increasing fatigue in November 2017, a few days after returning from a 2-week trip to California and Nevada, USA. She had not noticed any tick bites during the trip but had had several tick bites in Sweden during the summer of 2017. She subsequently started experiencing progressing difficulties with concentration and memory and had relapsing febrile episodes. In January 2018, she received diagnoses of uveitis and iritis; vitritis of unknown cause was later diagnosed. She also had progressive hearing loss, and hearing aids were prescribed. In addition, she had loss of appetite and weight (15 kg within 6 months). In May she had a short episode of left-sided weakness, and transient ischemic attack was suspected. All this resulted in her quitting her work as an accountant and hardly being able to leave her house.

Upon referral, we performed a lumbar puncture, which showed total CSF leukocytes of 331 cells/μL (reference <5 cells/μL), 273 cells/μL mononuclear (reference <5 cells/μL), albumin 1,550 mg/L (reference <420 mg/L), lactate 4.2 mmol/L (reference 1.2–2.1 mmol/L), and CXCL13 >500 pg/mL (reference <250 pg/mL). Magnetic resonance imaging showed contrast enhancement in both oculomotor nerves and the left trigeminal nerve, as well as thickening of the pituitary stalk. CSF was PCR negative for herpes simplex virus, varicella zoster virus, enterovirus, *Mycoplasma*, and *Toxoplasma*. Bacterial, mycobacterial, and fungal CSF cultures were negative. Serologic results for tickborne encephalitis and Lyme borreliosis were negative, with the exception of detectable *B. burgdorferi* IgM in CSF (Table). The *16S* rRNA gene sequencing (*9*) was positive for *B. miyamotoi*.

We treated the patient with doxycycline (200 mg 2×/d for 14 d); within 5 days, the patient regained her hearing, and the fever and headache disappeared. MRI 1 month later showed an almost complete regression of the contrast enhancement of the cranial nerves. By follow-up 2 months after finishing the treatment, the patient had resumed employment and felt almost completely recovered.

We performed *B. miyamotoi* quantitative PCR (qPCR) targeting the flagellin gene, slightly modified from Hovius et al. (*6*) (Appendix). The 2 successive CSF samples and 1 serum sample from patient A were positive by qPCR, as were the CSF and plasma samples from patient B (Table).

**Figure 2:**
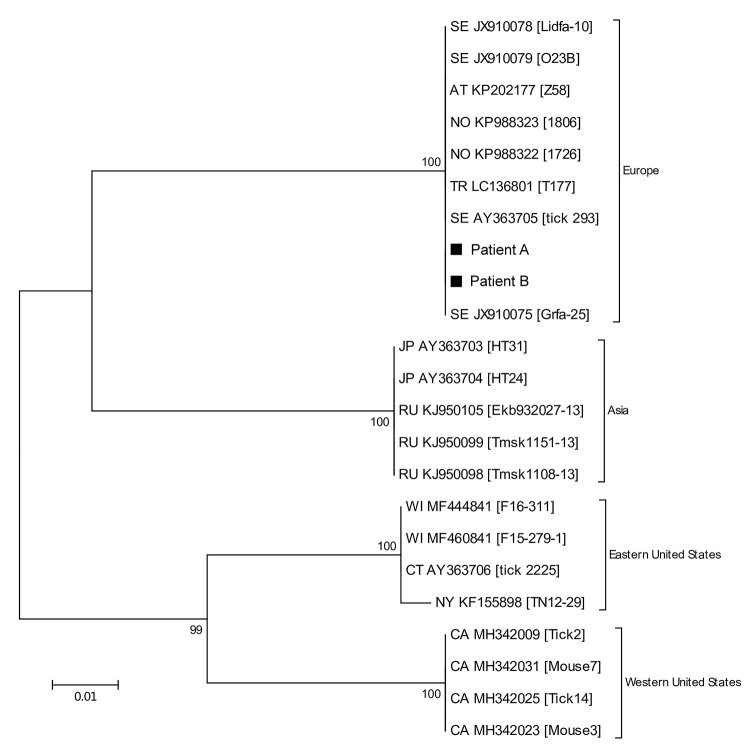
Phylogenetic tree based on 16S-23S intergenic spacer region sequences of *Borrelia miyamotoi* from 2 patients in Sweden, 2018 (patients A and B, black squares), and reference sequences. Tree constructed using the maximum-likelihood method based on the Tamura-Nei model and complete deletion. Sequences detected from patients in this study were deposited into GenBank under accession nos. MK458687 (patient A) and MK458688 (patient B). The source of each reference sequence is indicated by an accession number preceded by a state or country code: AT, Austria; CA, California; CT, Connecticut; JP, Japan; NO, Norway; NY, New York; RU, Russian Federation; SE, Sweden; TR, Turkey; WI, Wisconsin. The accession number is followed by the isolate name in brackets. The reliability of the tree was tested by 500 bootstrap replicate analyses; only values >50% are shown. The phylogenetic relationship between the *B. miyamotoi* strains detected in our patients was corroborated by the DNA sequences obtained from the *glpQ* and *p66* genes (data not shown). Scale bar indicates nucleotide substitutions per site.

From 1 of the CSF samples from patient A and the CSF sample from patient B, we confirmed the presence of *B. miyamotoi* by nested PCR amplification and sequencing of the glycerophosphodiester-phosphodiesterase (*glpQ)* and *p66* genes (*6*), as well as a fragment of the intergenic spacer (IGS) between the *16S* rRNA and *23S* rRNA genes (*10*) (Appendix). The DNA sequences of the *16S*-*23S* IGS (Figure 2), *glp*Q, and *p66* from patients A and B were identical to *B. miyamotoi* sequences derived from Europe but different from sequences derived from Asia and North America, indicating BMD contracted in Europe.

We tested for GlpQ and variable major proteins (Vmps) IgM and IgG by ELISA, as described previously (*11*,*12*) (Table). A clear seroconversion from IgM to IgG against GlpQ was demonstrated in patient A, whereas patient B merely demonstrated IgM reactivity against GlpQ. However, in patient A, but not in patient B (the immunosuppressed patient), we could demonstrate IgM and IgG against different Vmps over time.

Finally, we pursued culture attempts in MKP-F media on CSF and serum samples drawn before initiation of antimicrobial drug treatment, retrieved from −80°C (Table), as described by Koetsveld et al. (*13*). After 2 months of culture, all samples remained negative.

## Conclusions

Epidemiologic surveillance of emerging tickborne pathogens is crucial to increase awareness of the diseases that can be contracted after tick bites. Previous studies have shown that *B. miyamotoi* is present in *Ixodes ricinus* ticks in Scandinavia (*14*,*15*), but no human cases of BMD have been reported, and public health importance has been uncertain. Until now, severe disease, including slowly progressive CNS symptoms (*6*,*7*), has been reported in immunocompromised patients, but our findings indicate that *B. miyamotoi* may also cause CNS infection in immunocompetent persons (patient A). The clinical presentation differs from that of LNB, and results of serologic tests that are routinely used for LNB diagnosis can be negative. Therefore, we need to raise awareness of BMD among healthcare providers and ensure that adequate diagnostic methods are available. BMD should be a differential diagnosis in cases of fever and CNS symptoms after a tick bite in both immunosuppressed and immunocompetent persons.

Appendix Discussion of PCR protocols and serologic analyses used for the diagnosis of *Borrelia miyamotoi* in 2 patients in Sweden.
