# Supplementary material for: Two Cases of Borrelia miyamotoi Meningitis, Sweden, 2018
Source: Emerg Infect Dis. 2019 Oct;25(10):1965–8. doi: 10.3201/eid2510.190416 (PMC6759261; doi:10.3201/eid2510.190416)
Supplement: Appendix — Discussion of PCR protocols and serologic analyses used for the diagnosis of Borrelia miyamotoi in 2 patients in Sweden. [file 19-0416-Techapp-s1.pdf]

Article DOI: https://doi.org/10.3201/eid2510.190416

Two Cases of *Borrelia miyamotoi* Meningitis, Sweden, 2018

Appendix

PCR Protocols

16S Next Generation Sequencing (NGS) Analysis Using Ion Torrent Platform

A ≈460 bp region of the 16S rRNA gene covering the hypervariable regions V3–V4 was amplified using PCR primers containing adaptor sequences and barcodes for downstream Ion Torrent sequencing. Ion Torrent sequencing was performed according to a modified version of the manufacturer's protocol using an Ion S5 XL instrument (http://www.thermofisher.com). The 16S sequences obtained were mapped to curated 16S databases (https://www.arb-silva.de). All sequencing reactions contained an internal DNA control, facilitating analysis of very low DNA contents and also allowing semiquantitative aspects of the samples. The analysis was performed at the Public Health Agency of Sweden.

Borrelia miyamotoi qPCR

A qPCR targeting the *B. miyamotoi* flagellin, as described by Hovius et al. (*1*), was modified and performed using the a BioRad CFX96 Real-Time PCR System (Bio-Rad Laboratories, http://www.bio-rad.com). Optimized conditions in a final volume of 20 µL were Maxima Probe qPCR Mix, 200 nM forward (5′-AGA AGG TGC TCA AGC AG-3′) and reverse (5′-TCG ATC TTT GAA AGT GAC ATA T-3′) primers each, 200 nM probe (5′-FAM-AGC ACA ACA GGA GGG AGT TCA AGC- BHQ1–3′), 5 µL of template DNA and RNase-free water up to 20 µL. Cycling conditions were: 10 min 95°C followed by 45 cycles of 95°C for 5 s and 60°C for 35 s, and finally 1 cycle of 37°C for 20 s. Samples with a positive signal were assumed to be positive for *Borrelia miyamotoi*. The specificity of primers and probe was evaluated earlier by Hovius et al. (*1*). As a positive control, a synthetic plasmid containing the target sequence of the assay was used.

Molecular Identification/Typing of *Borrelia miyamotoi*

The presence of *B. miyamotoi* was confirmed by nested PCR amplification and sequencing of a fragment of the intergenic spacer between *16S* rRNA gene and *23S* rRNA gene, as described earlier by Bunikis et al. (*2*). The first reaction mixture in a final volume of 50 µl were: 5X Phusion HF Buffer (Thermo Scientific, http://www.thermofisher.com), 200 µM dNTP, 500 nM forward (5′-GTA TGT TTA GTG AGG GGG GTG-3′) and reverse (5′-GGA TCA TAG CTC AGG TGG TTA G-3′) primers each, 0.5 U Phusion DNA Polymerase (Thermo Scientific), 5 µL of template DNA and RNase-free water up to 50 µL. Cycling conditions were 5 min at 98°C followed by 39 cycles of 94°C for 30 s, 58°C for 30 s, and 74°C for 60 s, and finally 1 cycle of 74°C for 7 min. Five µL of PCR product obtained from the first reaction was added to a second reaction mixture in a final volume of 50 µL consisting of 5X Phusion HF Buffer, 200 µM dNTP, 500 nM nested-forward (5′-AGG GGG GTG AAG TCG TAA CAA G-3′) and nested-reverse (5′-GTC TGA TAA ACC TGA GGT CGG A-3′) primers each, 0.5 U Phusion DNA polymerase, and RNase-free water up to 50 µL. Cycling conditions were 5 min at 98°C followed by 40 cycles of 94°C for 30 s, 60°C for 30 s and 74°C for 60 s, and finally 1 cycle of 74°C for 7 min.

PCR amplification of the *glpQ* gene was performed as earlier described by Hovius et al. (*1*). The reaction mixture in a final volume of 25 µL were 5X Phusion HF Buffer (Thermo Scientific), 200 µM dNTP, 500 nM forward (5′-ATG GGT TCA AAC AAA AAG TCA ML-3′) and reverse (5′-CAT TAC TGT GTC AGT AAA ATC TGT AAA TAT ACC ATC TAC-3′) primers each, 0.5 U Phusion DNA Polymerase (Thermo Scientific), 2.5 µL of template DNA, and RNase-free water up to 25 µL. Cycling conditions were 15 min at 94°C, then cycles of 20 s at 94°C, 30 s at 70°C, 30 s at 72°C lowering annealing temperature 1°C each cycle to 60°C, then 40 cycles at this annealing temperature and a final extension step of 7 min at 72°C.

The *p66* gene was amplified under identical conditions with different forward (5′-GAT ACT AAA TTA TTA AAT CCA AAA TCG-3′) and reverse (5′-GGA AAT GAG TAC CTA CAT ATG G-3′) primers, as described earlier by Hovius et al. (*1*). Nucleotide sequencing of the PCR products was performed by Macrogen Inc. (Amsterdam; https://macrogenlab.com). All sequences were confirmed by sequencing both strands. The obtained chromatograms were edited and analyzed using BioEdit software v7.0 (Tom Hall, Ibis Therapeutics,

https://bioedit.software.informer.com). Sequence alignment of the sequences acquired in this study and a representative selection of *B. miyamotoi* 16S-23S IGS, *glpQ*, and *p66* sequences (detected in different specimens; *I. ricinus, I. pacificus, I. scapularis, I. persulcatus* and human isolates in Europe, Asia, and North America) deposited in GenBank was performed using BioEdit. Phylogenetic analyses were conducted using MEGA version 7 (https://www.megasoftware.net), and the phylogenetic tree of 16S–23S IGS sequences was constructed by using the maximum likelihood method based on the Tamura-Nei model with complete deletion. The significance of the relationship was ascertained by bootstrap analysis (500 replicates). Sequences obtained in this investigation have been deposited in GenBank with accession numbers MK458687–MK458692.

Serologic Analyses

GlpQ and Vmps ELISA

The presence of anti-glycerophosphodiester-phosphodiesterase (GlpQ) and anti-Variable major proteins (Vmps) IgM and IgG antibodies was analyzed by ELISA, as described previously (*3*). Positive and negative controls were included in each run and the cutoff (optical density, OD) for reactivity was set at the median + 4 x SD of 8 healthy controls from a non-endemic area (northern Norway). We have recently shown that Vmps are highly immunogenic in patients with BMD and that the presence of antibodies against GlpQ combined with antibodies against Vmps had a 100% specificity for IgM and 98.3% for IgG (*4*).

References

1. Hovius JW, de Wever B, Sohne M, Brouwer MC, Coumou J, Wagemakers A, et al. A case of meningoencephalitis by the relapsing fever spirochaete *Borrelia miyamotoi* in Europe. Lancet. 2013;382:658. https://doi.org/10.1016/S0140-6736(13)61644-X

2. Bunikis J, Garpmo U, Tsao J, Berglund J, Fish D, Barbour AG. Sequence typing reveals extensive strain diversity of the Lyme borreliosis agents *Borrelia burgdorferi* in North America and *Borrelia afzelii* in Europe. Microbiology. 2004;150:1741–55. https://doi.org/10.1099/mic.0.26944-0

3. Wagemakers A, Koetsveld J, Narasimhan S, Wickel M, Deponte K, Bleijlevens B, et al. Variable major proteins as targets for specific antibodies against *Borrelia miyamotoi*. J Immunol. 2016;196:4185–95. https://doi.org/10.4049/jimmunol.1600014

4. Koetsveld J, Kolyasnikova NM, Wagemakers A, Stukolova OA, Hoornstra D, Sarksyan DS, et al. Serodiagnosis of *Borrelia miyamotoi* disease by measuring antibodies against GlpQ and variable major proteins. Clin Microbiol Infect. 2018;24:1338.e1–7. https://doi.org/10.1016/j.cmi.2018.03.009
